# Population Attributable Fractions for Risk Factors for Dementia in Latin America

**DOI:** 10.1002/alz.091861

**Published:** 2025-01-09

**Authors:** Regina Silva Paradela, Ismael Luis Calandri, Natalia Pozo, Emanuel Garat, Carolina Delgado, Lucia Crivelli, Kristine Yaffe, Cleusa P Ferri, Naaheed Mukadam, Gill Livingston, Claudia Kimie Suemoto

**Affiliations:** ^1^ Global Brain Health Institute, Memory and Aging Center, University of California San Francisco, San Francisco, CA USA; ^2^ University of São Paulo Medical School, São Paulo, São Paulo Brazil; ^3^ Fleni, Buenos Aires, Buenos Aires Argentina; ^4^ Hospital Clínico Universidad de Chile, Santiago Chile; ^5^ Hospital San Borja Arriarán, Santiago Chile; ^6^ Universidad de Chile, Santiago, Region Metropolitana Chile; ^7^ Hospital Clínico Universidad de Chile, Santiago, Region Metropolitana Chile; ^8^ Department of Cognitive Neurology, Montañeses, Buenos Aires Argentina; ^9^ Departments of Psychiatry and Behavioral Sciences, Neurology, and Epidemiology, University of California San Francisco, San Francisco, CA USA; ^10^ Hospital Alemão Oswaldo Cruz, São Paulo, São Paulo Brazil; ^11^ Federal University of Sao Paulo (UNIFESP), Sao Paulo Brazil; ^12^ University College London, London United Kingdom

## Abstract

**Background:**

Approximately 40% of global dementia cases are associated with 12 modifiable risk factors (less education, hearing loss, hypertension, obesity, smoking, depression, social isolation, physical inactivity, diabetes, alcohol excess, air pollution, and traumatic brain injury). However, the number of people with these risk factors differs between populations. Latin American countries differ in socioeconomic and geographic aspects and, therefore, risk factors prevalence. Our goal was to determine the population‐attributable fraction (PAF) of these risk factors (combined and individually) for dementia by analyzing population‐based samples from seven countries in Latin America (LA).

**Method:**

We used data from Argentinian, Brazilian, Bolivian, Chilean, Honduran, Mexican, and Peruvian cross‐sectional, nationally representative surveys with harmonized measurements of the 12 modifiable risk factors for dementia. Data were collected between 2015 and 2021, and the survey sample size varied from 5,995 to 107,907 participants aged 18 years or older across the seven countries. We calculated the risk factor prevalence and their communalities in each country and used these with relative risks for dementia from previous meta‐analyses to calculate the PAFs. Pooled PAFs for LA were then obtained using random effect meta‐analyses.

**Result:**

The overall proportion of dementia cases attributed to 12 modifiable risk factors varied across LA countries (62% in Chile, 60% in Argentina, 56% in Bolivia, 56% in Mexico, 54% in Honduras, 48% in Brazil, and 45% in Peru), with an overall PAF of 54% for LA (95% CI 48.8‐59.6). Obesity (weighted PAF = 7%), physical inactivity (weighted PAF = 6%), and depression (weighted PAF = 5%) had the highest PAFs in LA.

**Conclusion:**

There is potential for prevention of a higher proportion of dementia in LA than worldwide. Obesity, physical inactivity, and depression are the major contributors to dementia variance across the seven LA countries. These findings have implications for dementia prevention strategies in LA.

